# Supplementary material for: Improved unsupervised physics‐informed deep learning for intravoxel incoherent motion modeling and evaluation in pancreatic cancer patients
Source: Magn Reson Med. 2021 Jun 9;86(4):2250–65. doi: 10.1002/mrm.28852 (PMC8362093; doi:10.1002/mrm.28852)
Supplement: Supplementary file 1 — FIGURE S1 Plots of the estimated IVIM parameters (D, f, D*) where no Spearman rank correlation coefficient (ρ) can be determined and is set to a ρ of 1. In all plots, the values of the simulations are presented in grey FIGURE S2 Normalized root‐mean‐square error (NRMSE) boxplots of the estimated IVIM parameters (D, f, D*) that contain all hyperparameter combinations with a fixed learning rate set to 1 × 10^−4^ and a fixed number of hidden layers set to 3 at SNR 20 for 50 repeated trainings. Highlighted in green is the intermediate step of IVIM‐NEToptim. Left of each plot shows the LS approach (blue), Bayesian approach (brown) and IVIM‐NETorig (orange; LR = 1 × 10^−3^) FIGURE S3 Spearman rank correlation coefficient (ρ) boxplots of the estimated IVIM parameters (D, f, D*) that contain all hyperparameter combinations with a fixed learning rate set to 1 × 10^−4^ and a fixed number of hidden layers set to 3 at SNR 20 for 50 repeated trainings. Highlighted in green is the intermediate step of IVIM‐NEToptim. Left of each plot shows the LS approach (blue), Bayesian approach (brown) and IVIM‐NETorig (orange; LR = 1 × 10^−3^) FIGURE S4 Normalized Coefficient of variation (CVNET) plots of the estimated IVIM parameters (D, f, D*) that contain all hyperparameter combinations with a fixed learning rate set to 1 × 10^−4^ and a fixed number of hidden layers set to 3 at SNR 20 for 50 repeated trainings. Highlighted in green is the intermediate step of IVIM‐NEToptim. Left of each plot shows the LS approach (blue), Bayesian approach (brown) and IVIM‐NETorig (orange; LR = 1 × 10^−3^) FIGURE S5 Ranked plots of the metrics (NRMSE, ρ and CVNET) of evaluation 1 that contain all hyperparameter combinations with a fixed learning rate set to 1 × 10^−4^ and a fixed number of hidden layers set to 3 at SNR 20 for 50 repeated trainings. Highlighted in green is the intermediate step of IVIM‐NEToptim. Left of each plot shows the LS approach (blue), Bayesian approach (brown) and IVIM‐NETorig (orange; LR = 1 × 10 [file MRM-86-2250-s001.docx]

#### Supporting Information 1: Simulations

####
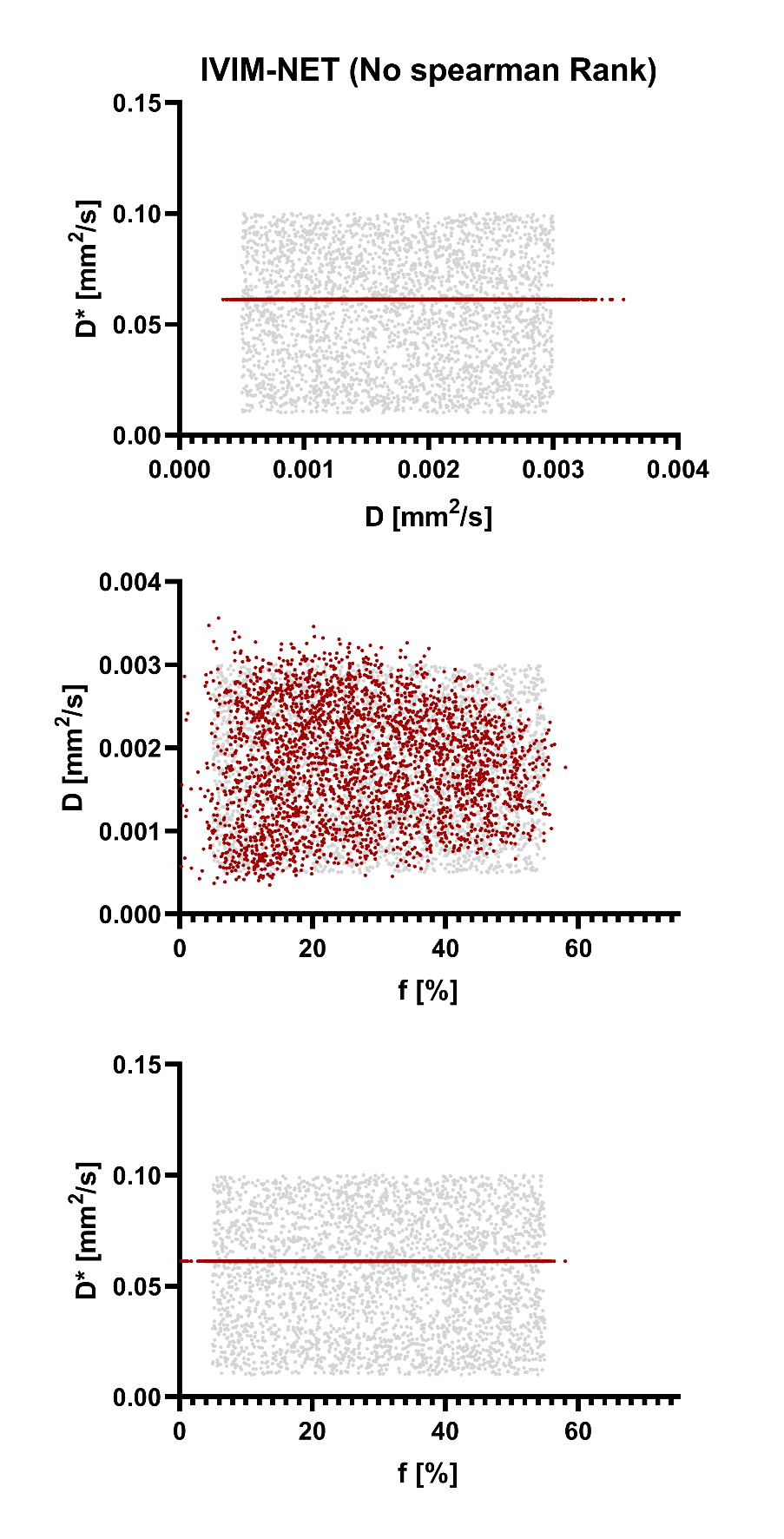


Figure S1: Plots of the estimated IVIM parameters (*D*, *f*, *D**) where no Spearman rank correlation coefficient (*ρ*) can be determined and is set to a *ρ* of 1. In all plots, the values of the simulations are presented in grey.


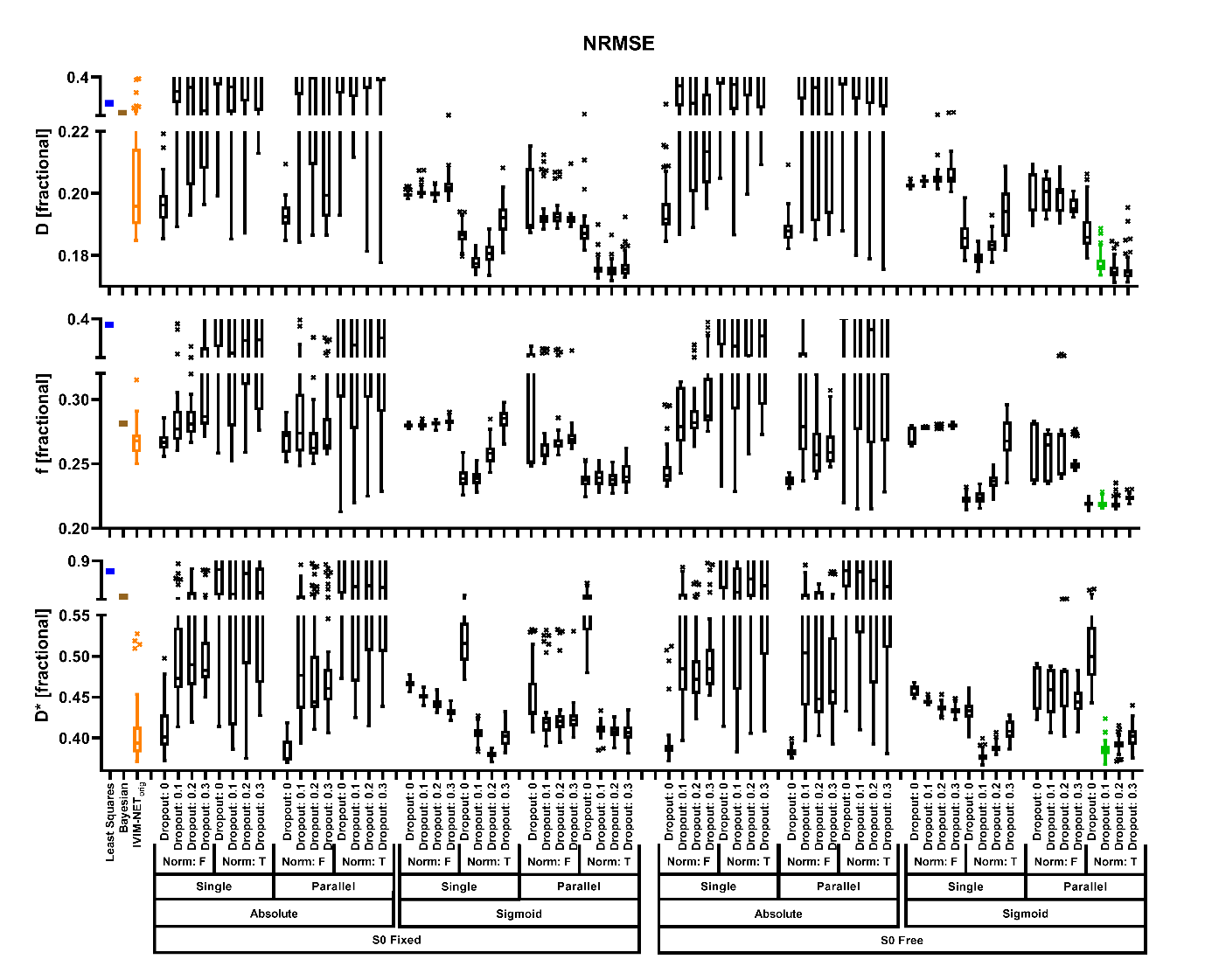


Figure S2: Normalized root-mean-square error (NRMSE) boxplots of the estimated IVIM parameters (*D*, *f*, *D**) that contain all hyperparameter combinations with a fixed learning rate set to 1 × 10^-4^ and a fixed number of hidden layers set to 3 at SNR 20 for 50 repeated trainings. Highlighted in green is the intermediate step of IVIM-NET_optim_. Left of each plot shows the LS approach (blue), Bayesian approach (brown) and IVIM-NET_orig_ (orange; LR = 1 × 10^-3^).


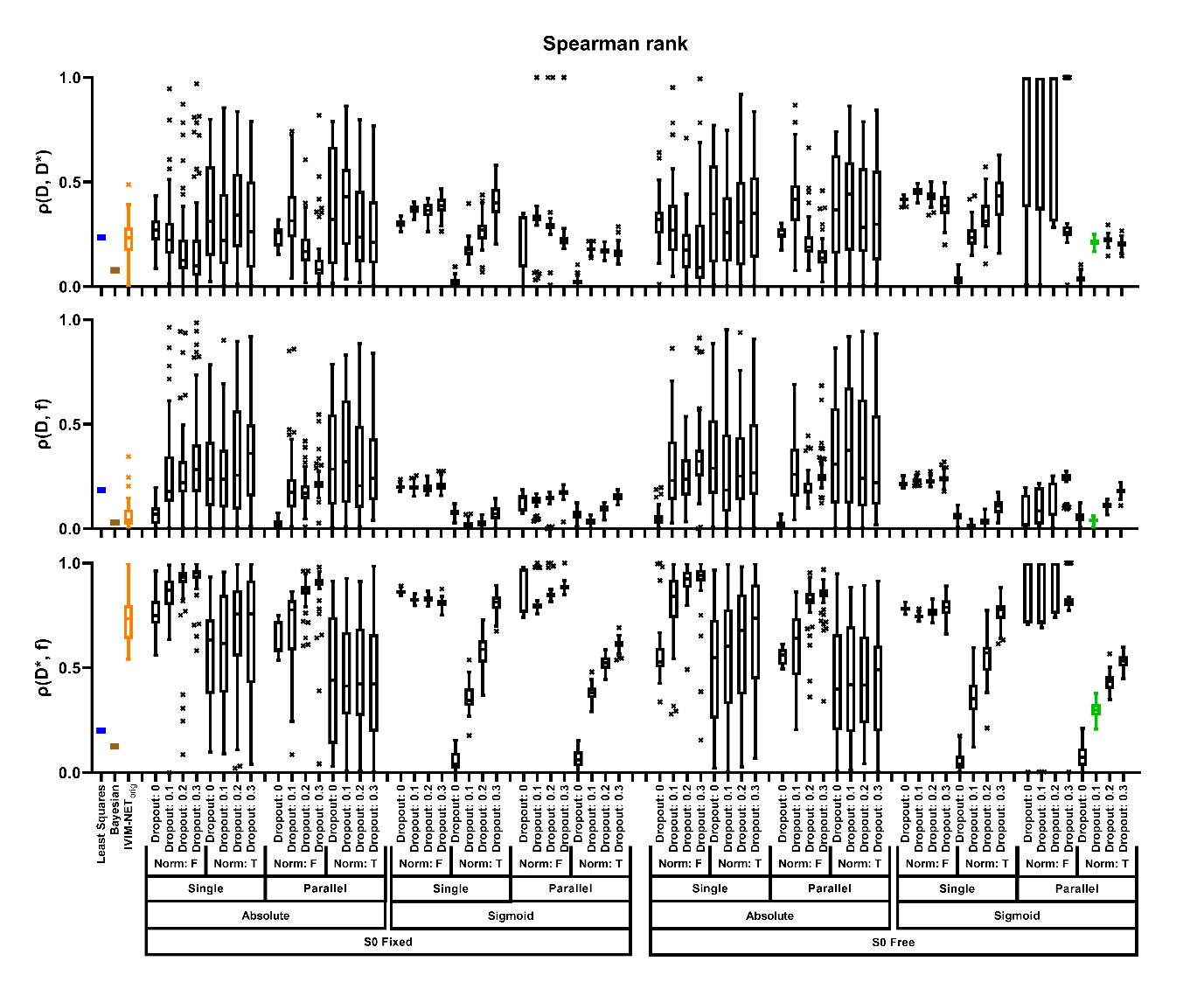


Figure S3: Spearman rank correlation coefficient (*ρ*) boxplots of the estimated IVIM parameters (*D*, *f*, *D**) that contain all hyperparameter combinations with a fixed learning rate set to 1 × 10^-4^ and a fixed number of hidden layers set to 3 at SNR 20 for 50 repeated trainings. Highlighted in green is the intermediate step of IVIM-NET_optim_. Left of each plot shows the LS approach (blue), Bayesian approach (brown) and IVIM-NET_orig_ (orange; LR = 1 × 10^-3^).


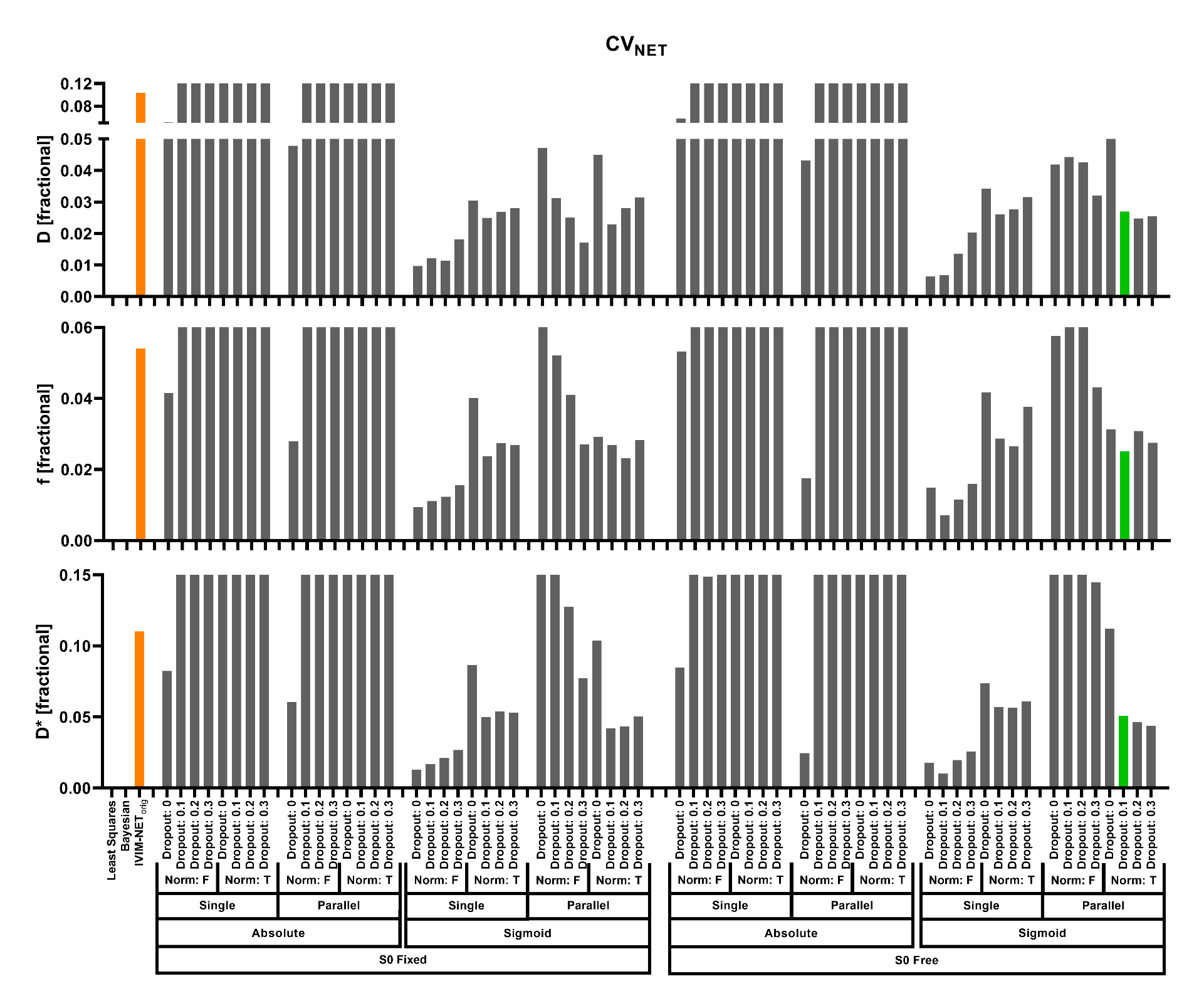


Figure S4: Normalized coefficient of variation (CV_NET_) plots of the estimated IVIM parameters (*D*, *f*, *D**) that contain all hyperparameter combinations with a fixed learning rate set to 1 × 10^-4^ and a fixed number of hidden layers set to 3 at SNR 20 for 50 repeated trainings. Highlighted in green is the intermediate step of IVIM-NET_optim_. Left of each plot shows the LS approach (blue), Bayesian approach (brown) and IVIM-NET_orig_ (orange; LR = 1 × 10^-3^). As the LS and Bayesian approaches are deterministic, their CV_NET_ was zero and not plotted.


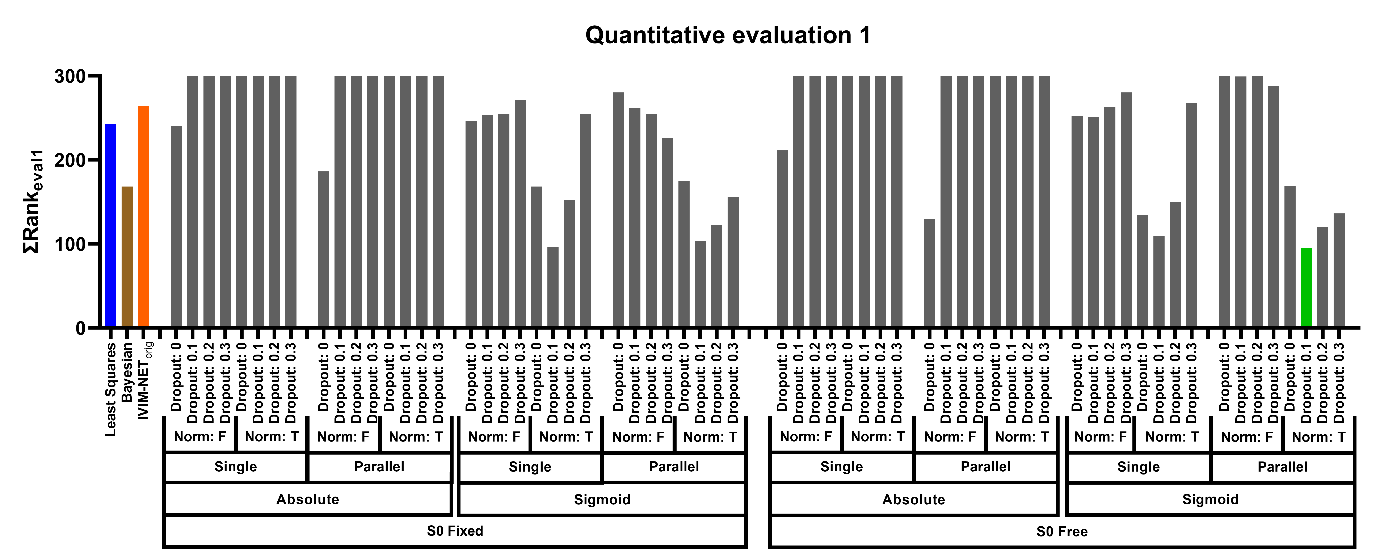


Figure S5: Ranked plots of the metrics (NRMSE, *ρ* and CV_NET_) of evaluation 1 that contain all hyperparameter combinations with a fixed learning rate set to 1 × 10^-4^ and a fixed number of hidden layers set to 3 at SNR 20 for 50 repeated trainings. Highlighted in green is the intermediate step of IVIM-NET_optim_. Left of each plot shows the LS approach (blue), Bayesian approach (brown) and IVIM-NET_orig_ (orange; LR = 1 × 10^-3^).


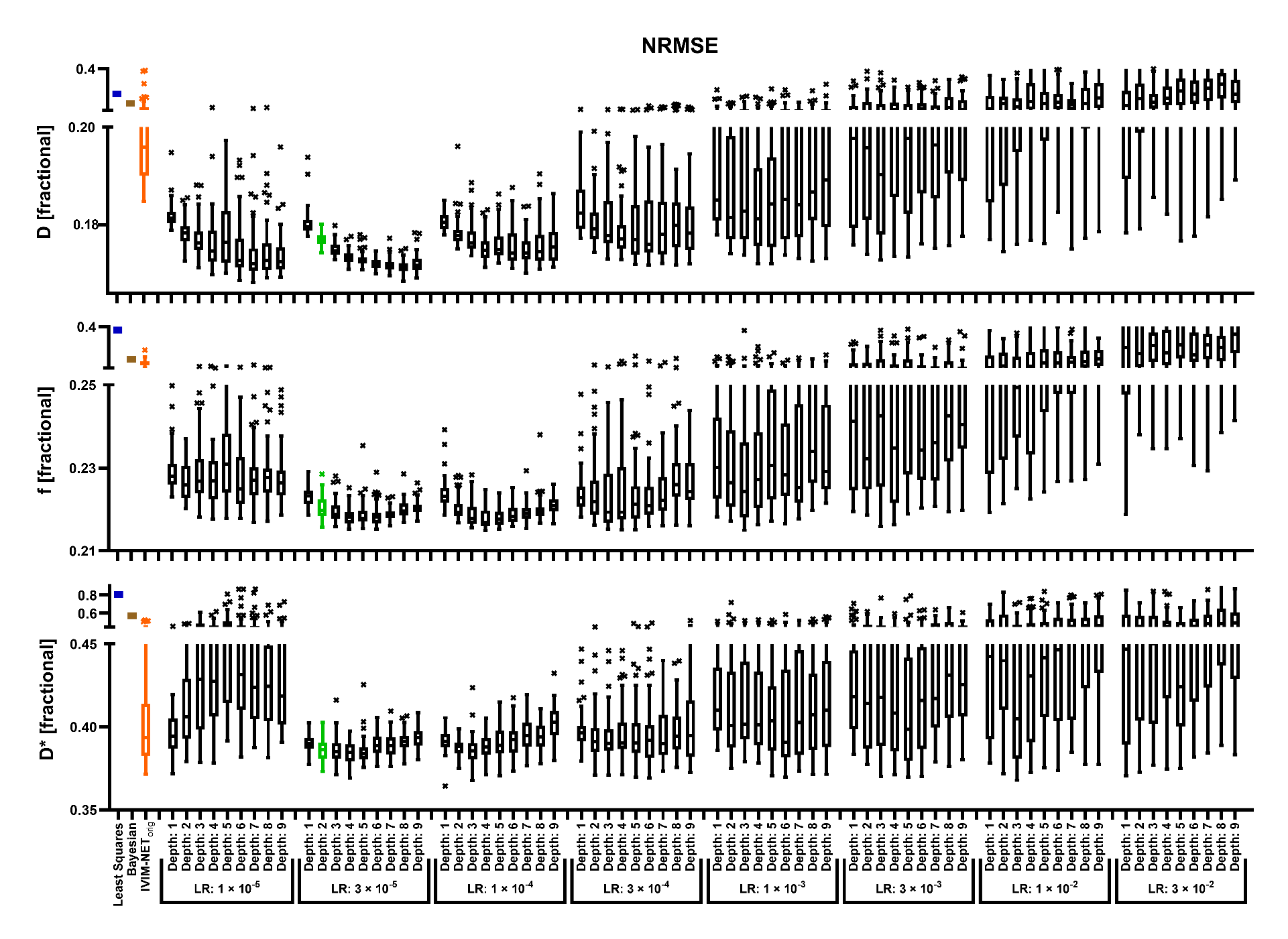


Figure S6: Normalized root-mean-square error (NRMSE) boxplots of the estimated IVIM parameters (*D*, *f*, *D**) of the second evaluation for different LR and number of hidden layers, with fixed hyperparameters of extra fitting parameter S0, sigmoid activation functions, a parallel network architecture, 10% dropout and batch normalization at SNR 20 for 50 repeated trainings. Highlighted in green is IVIM-NET_optim_. Left of each plot shows the LS approach (blue) and Bayesian approach (brown) and IVIM-NET_orig_ (orange).


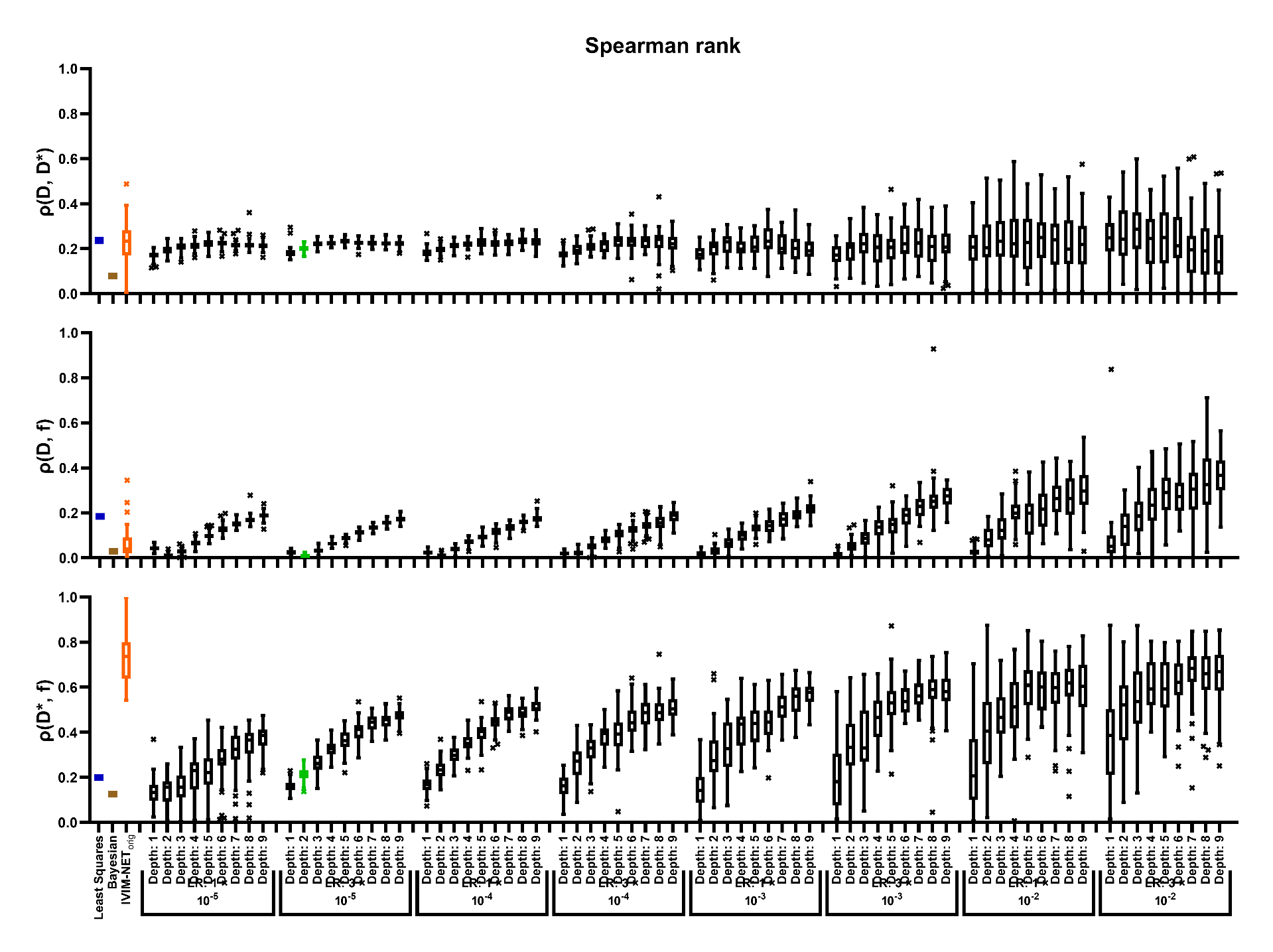


Figure S7: Spearman rank correlation coefficient (*ρ*) boxplots of the estimated IVIM parameters (*D*, *f*, *D**) of the second evaluation for different LR and number of hidden layers, with fixed hyperparameters of extra fitting parameter S0, sigmoid activation functions, a parallel network architecture, 10% dropout and batch normalization at SNR 20 for 50 repeated trainings. Highlighted in green is IVIM-NET_optim_. Left of each plot shows the LS approach (blue) and Bayesian approach (brown) and IVIM-NET_orig_ (orange).


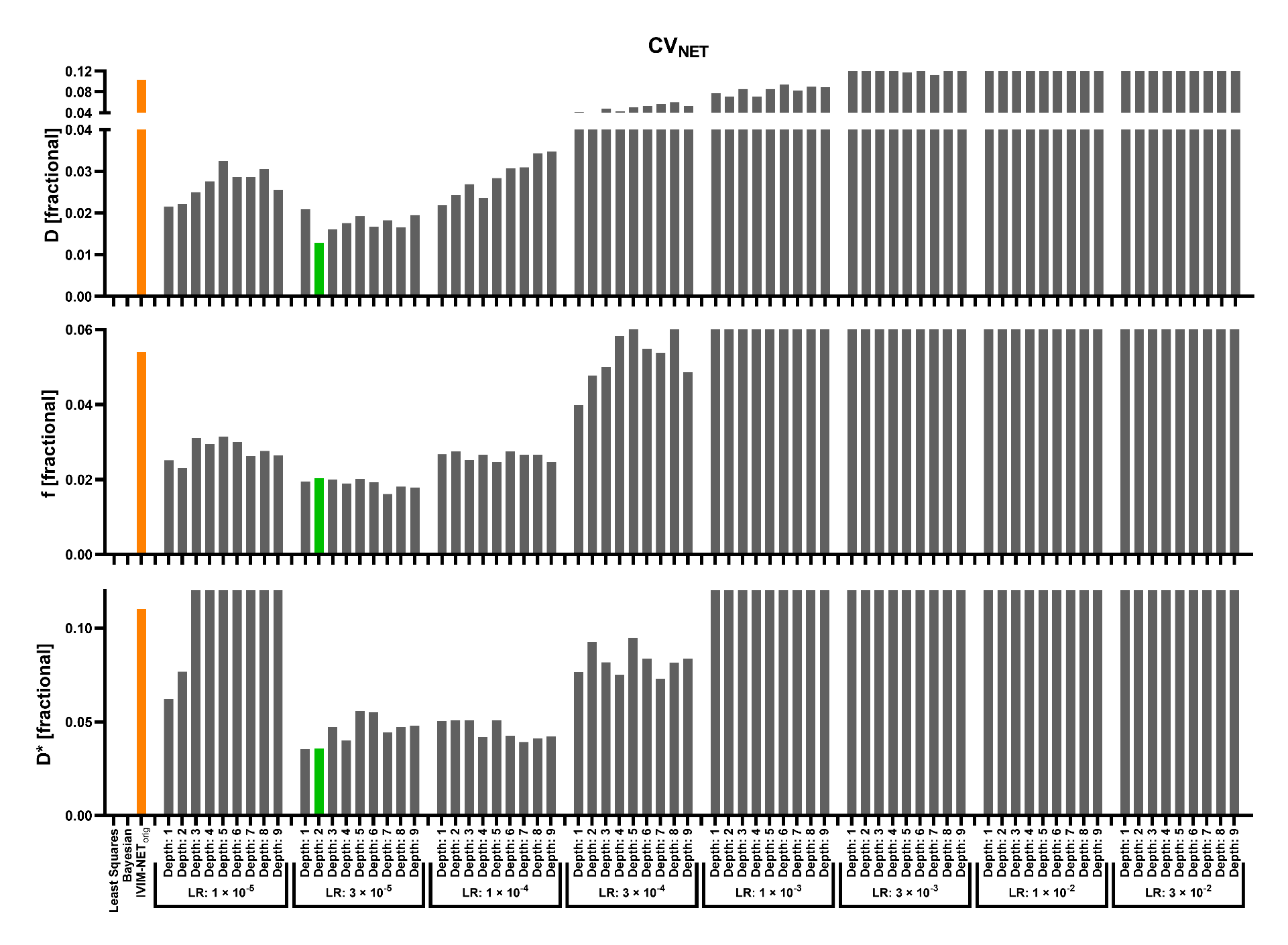


Figure S8: Normalized coefficient of variation (CV_NET_) plots of the estimated IVIM parameters (*D*, *f*, *D**) of the second evaluation for different LR and number of hidden layers, with fixed hyperparameters of extra fitting parameter S0, sigmoid activation functions, a parallel network architecture, 10% dropout and batch normalization at SNR 20 for 50 repeated trainings. Highlighted in green is IVIM-NET_optim_. Left of each plot shows the LS approach (blue) and Bayesian approach (brown) and IVIM-NET_orig_ (orange). As the LS and Bayesian approaches are deterministic, their CV_NET_ was zero and not plotted.


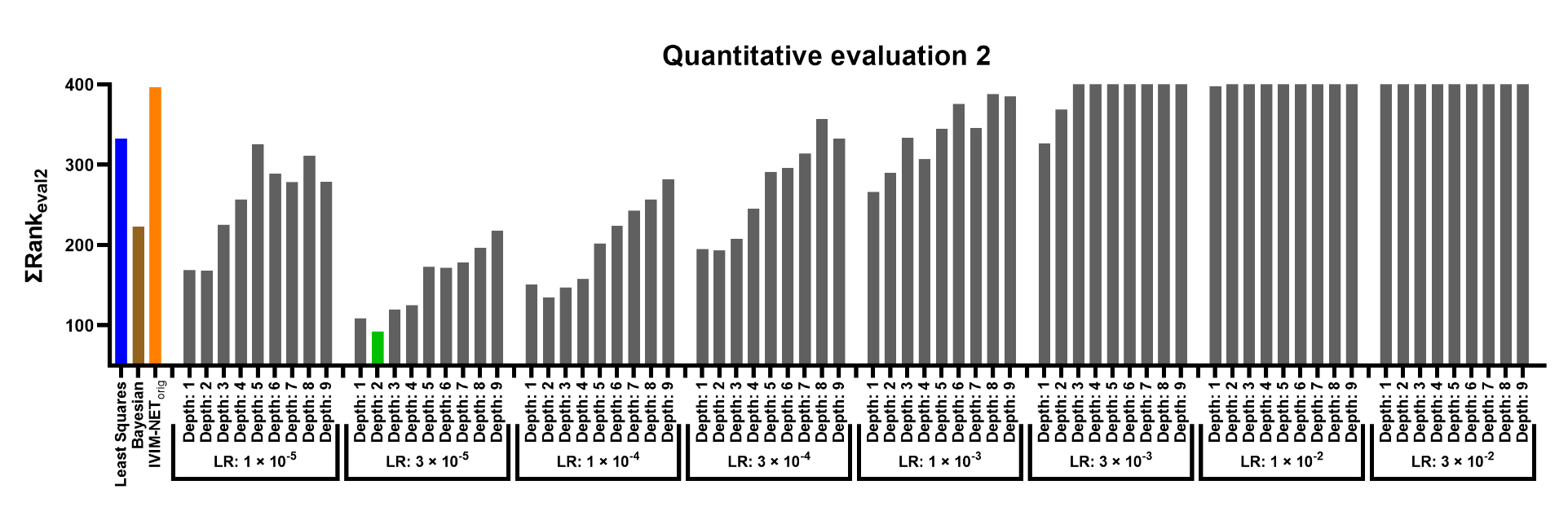


Figure S9: Ranked plots of the metrics (NRMSE, *ρ* and CV_NET_) of evaluation 2 for different LR and number of hidden layers, with fixed hyperparameters of extra fitting parameter S0, sigmoid activation functions, a parallel network architecture, 10% dropout and batch normalization at SNR 20 for 50 repeated trainings. Highlighted in green is IVIM-NET_optim_. Left of each plot shows the LS approach (blue) and Bayesian approach (brown) and IVIM-NET_orig_ (orange).

**
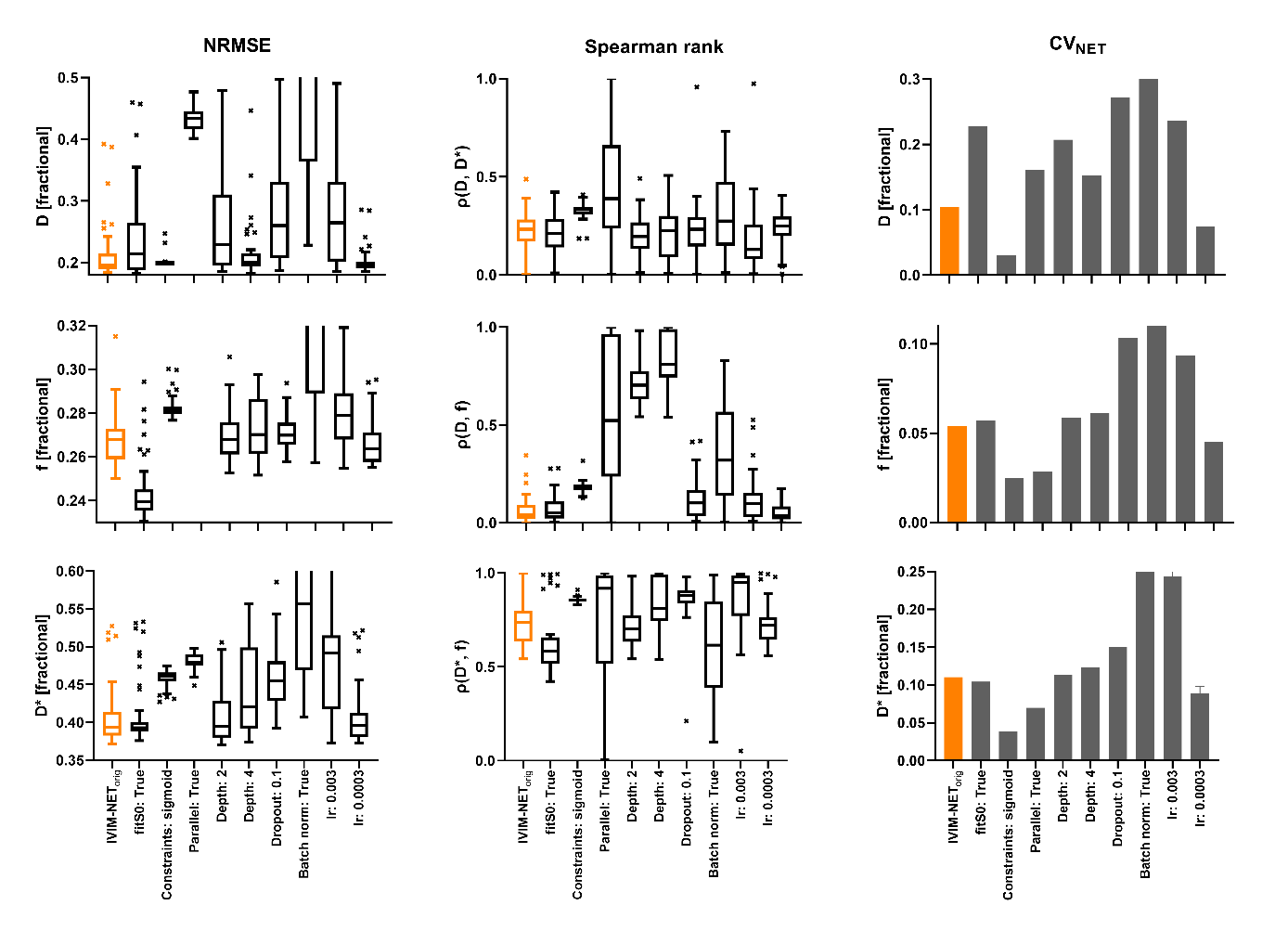
**

Figure S10: Normalised root-mean-square error (NRMSE; left), Spearman rank correlation coefficient (*ρ*; center) and normalized coefficient of variation (CV_NET_; right) plots of the estimated IVIM parameters (*D*, *f* and *D**) with a single parameter change for IVIM-NET_orig_ (orange) at SNR 20 for 50 repeated trainings. The *ρ(D*,f)* remains substantial for single deviations from IVIM-NET_orig_.

#### Supporting Information 2: Verification in patients with PDAC

For simplicity, we have made a table overview (Table S1) of every parameter maps of the Supporting Information. As in Figures 5 and 6 of our manuscript, these Supporting Information Figures S11-S20 show ‘IVIM parameter maps (*D*, *f*, *D**) of the LS approach, Bayesian approach and IVIM-NET_optim_ of a PDAC patient of the treated cohort before CRT or the test-retest cohort. The red ROI represents the PDAC and the green ROI represents homogenous 2D liver tissue ROI. The two highlighted blue regions correlate to the voxels from the plots below. The yellow square zooms in on the two highlighted voxels. In the plots, the small light grey dots are the repeated measures and the big black dots are the root-mean-squares of these repeated measures. The plot parameters are shown below.’

Table S1: Overview of the parameter maps of Supporting Information Figures S11-S20.

| Figure | Remark |  |
| --- | --- | --- |
| S11 | Highlighted voxels in the liver. The light blue voxel (left plot) shows consistency in IVIM parameters between the LS approach and IVIM-NET_optim_ with low *f,* and moderate *D* and *D**, while the Bayesian approach shows higher *f,* lower *D* and very low *D**. The neighboring dark blue voxel (right plot) shows no diffusion (*D* = 0 mm^2^/s), a very high *f* and very low *D** for the LS and Bayesian approaches. IVIM-NET_optim_ shows more consistency in IVIM parameters between the two neighboring voxels. The LS and Bayesian approaches show noisier parameter maps, particularly in the liver and around the tumor region. |  |
| S12 | Highlighted voxels in the tumor. The light blue voxel (left plot) shows indifferent IVIM parameters for all three fitting approaches. Although the data is similar in the neighboring dark blue voxel (right plot), the LS and Bayesian approaches compute a higher *f,* lower *D* (with *D* = 0 mm^2^/s for the LS approach) and very low *D** (to the lower bound of *D** = 5.0 × 10^-3^ mm^2^/s for the LS approach) compared to their parameters in the light blue voxel. IVIM-NET_optim_ shows more consistency in IVIM parameters between the two neighboring voxels. The LS and Bayesian approaches show noisier parameter maps, particularly in the liver and around the tumor region. Note that there is an artifact, which can be seen best in the middle part of the liver. |  |
| S13 | Highlighted voxels in the liver. The light blue voxel (left plot) shows consistency in IVIM parameters for all three fitting approaches with a high IVIM effect. Although the data is similar in the neighboring dark blue voxel (right plot), the LS and Bayesian approaches compute a higher *f,* and lower *D* and *D** compared to their parameters in the light blue voxel. IVIM-NET_optim_ shows more consistency in IVIM parameters between the two neighboring voxels. The LS and Bayesian approaches show noisier parameter maps, particularly in the liver and around the tumor region. |  |
| S14 | Highlighted voxels in the tumor. The light blue voxel (left plot) shows consistency in IVIM parameters for all three fitting approaches. Although the data is similar in the neighboring dark blue voxel (right plot), the LS and Bayesian approaches compute a higher *f,* and lower *D* and very low *D** (to the lower bound of *D** = 5.0 × 10^-3^ mm^2^/s for the LS approach) compared to their parameters in the light blue voxel. IVIM-NET_optim_ shows more consistency in IVIM parameters between the two neighboring voxels. The LS and Bayesian approaches show noisier parameter maps, particularly in the liver, kidneys and around the tumor region. |  |
| S15 | Highlighted voxels in the tumor. The light blue voxel (left plot) shows consistency in IVIM parameters between the LS approach and IVIM-NET_optim_ with low *f,* and moderate *D* and *D**, while the Bayesian approach shows higher *f,* lower *D* and very low *D**. Although the data is similar in the neighboring dark blue voxel (right plot), the LS and Bayesian approaches compute a higher *f,* and lower *D* and very low *D** (to the lower bound of *D** = 5.0 × 10^-3^ mm^2^/s) compared to their parameters in the light blue voxel. IVIM-NET_optim_ shows more consistency in IVIM parameters between the two neighboring voxels. The LS and Bayesian approaches show noisier parameter maps, particularly in the kidneys and around the tumor region. |  |
| S16 | Highlighted voxels in the tumor. The light blue voxel (left plot) shows consistency in IVIM parameters for all three fitting approaches. Although the data is similar in the neighboring dark blue voxel (right plot) with a lower IVIM effect, the LS and Bayesian approaches compute a higher *f,* and lower *D* and very low *D** (to the lower bound of *D** = 5.0 × 10^-3^ mm^2^/s) compared to their parameters in the light blue voxel. IVIM-NET_optim_ shows more consistency in IVIM parameters between the two neighboring voxels with a lower *f*. The LS and Bayesian approaches show noisier parameter maps, particularly around the tumor region. Note that the LS approach has a very high *D** (to the upper bound of *D** = 200 × 10^-3^ mm^2^/s) in the blue voxel, while in the neighboring dark blue voxel it has a very low *D** (to the lower bound of *D** = 5.0 × 10^-3^ mm^2^/s)*.* |  |
| S17 | Highlighted voxels in the liver. The light blue voxel (left plot) shows consistency in IVIM parameters for all three fitting approaches with a high IVIM effect. Although the data is similar in the neighboring dark blue voxel (right plot) with a lower IVIM effect, the LS and Bayesian approaches compute a higher *f,* and lower *D* and *D** compared to their parameters in the light blue voxel. IVIM-NET_optim_ shows more consistency in IVIM parameters between the two neighboring voxels with a lower *f*. The LS and Bayesian approaches show noisier parameter maps, particularly in the liver. |  |
| S18 | Highlighted voxels in the kidneys. The light blue voxel (left plot) shows consistency in IVIM parameters for all three fitting approaches. Although the data is similar in the neighboring dark blue voxel (right plot) with a lower IVIM effect, the Bayesian approaches compute a higher *f,* and lower *D* and very low *D** compared to its parameters in the light blue voxel. IVIM-NET_optim_ and the LS approach show more consistency in IVIM parameters between the two neighboring voxels with a lower *f*. The LS and Bayesian approaches show noisier parameter maps, particularly in the liver, kidneys and around the tumor region. |  |
| S19 | Highlighted voxels in the liver. The light blue voxel (left plot) shows consistency in IVIM parameters between the LS approach and IVIM-NET_optim_ with low *f,* and moderate *D* and *D**, while the Bayesian approach shows higher *f,* lower *D* and very low *D**. Although the data is similar in the neighboring dark blue voxel (right plot), the LS and Bayesian approaches compute a higher *f,* and lower *D* and very low *D** compared to their parameters in the light blue voxel. IVIM-NET_optim_ shows more consistency in IVIM parameters between the two neighboring voxels. The LS and Bayesian approaches show noisier parameter maps, particularly in the liver. |  |
| S20 | Highlighted voxels in the liver. The light blue voxel (left plot) shows consistency in IVIM parameters for all three fitting approaches. Although the data is similar in the neighboring dark blue voxel (right plot) with a lower IVIM effect, the Bayesian approaches compute a higher *f,* and lower *D* and very low *D** compared to its parameters in the light blue voxel. IVIM-NET_optim_ and the LS approach show more consistency in IVIM parameters between the two neighboring voxels with a lower *f*. The LS approach shows noisier parameter maps, particularly in the liver, kidneys and around the tumor region. Note that although the LS approach has a very high D* (to the upper bound of *D** = 200 × 10^-3^ mm^2^/s) in both voxels, the LS and IVIM-NET_optim_ show the same plots. |  |
|  | | |


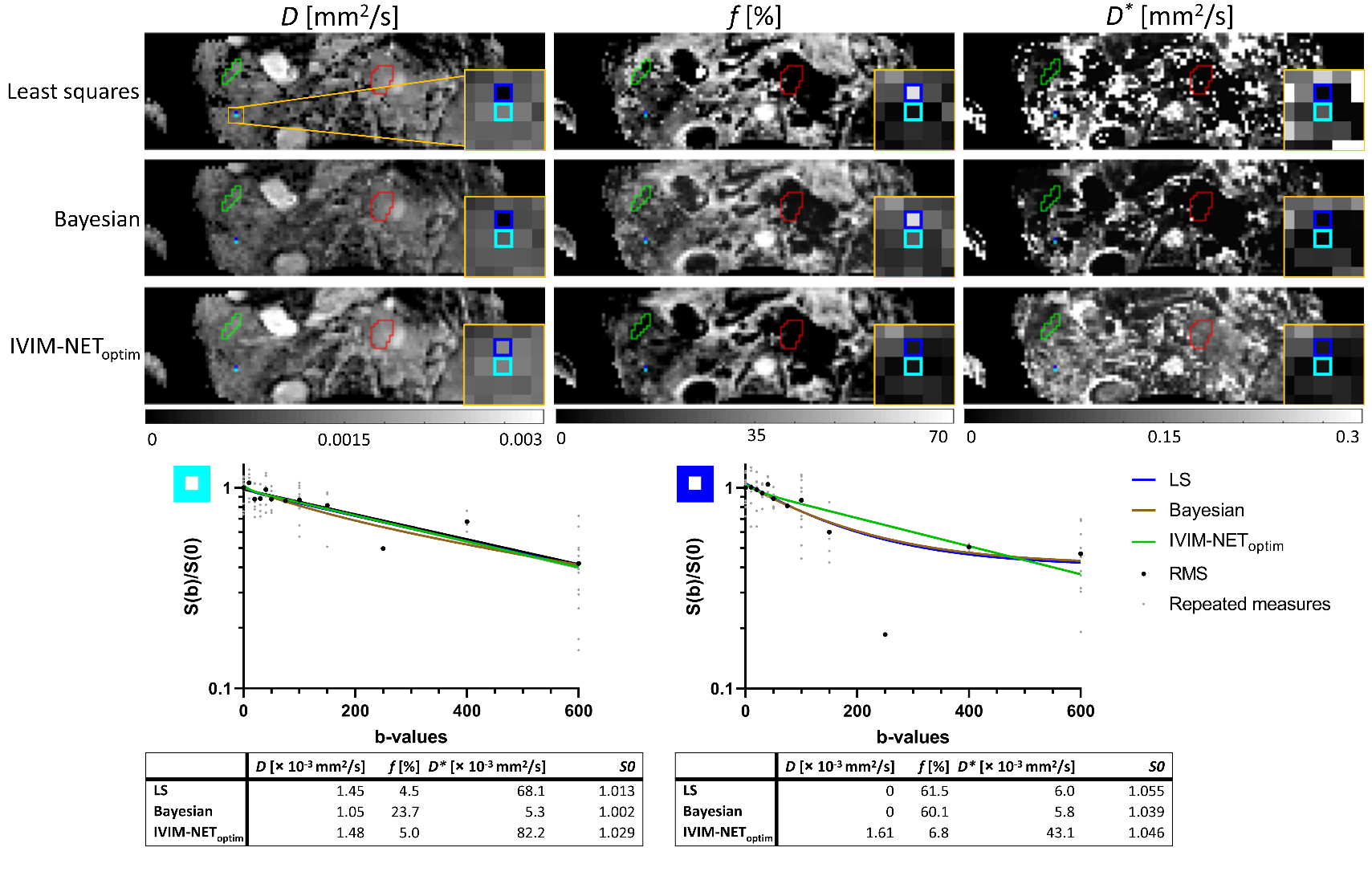


Figure S11: See Table S1.


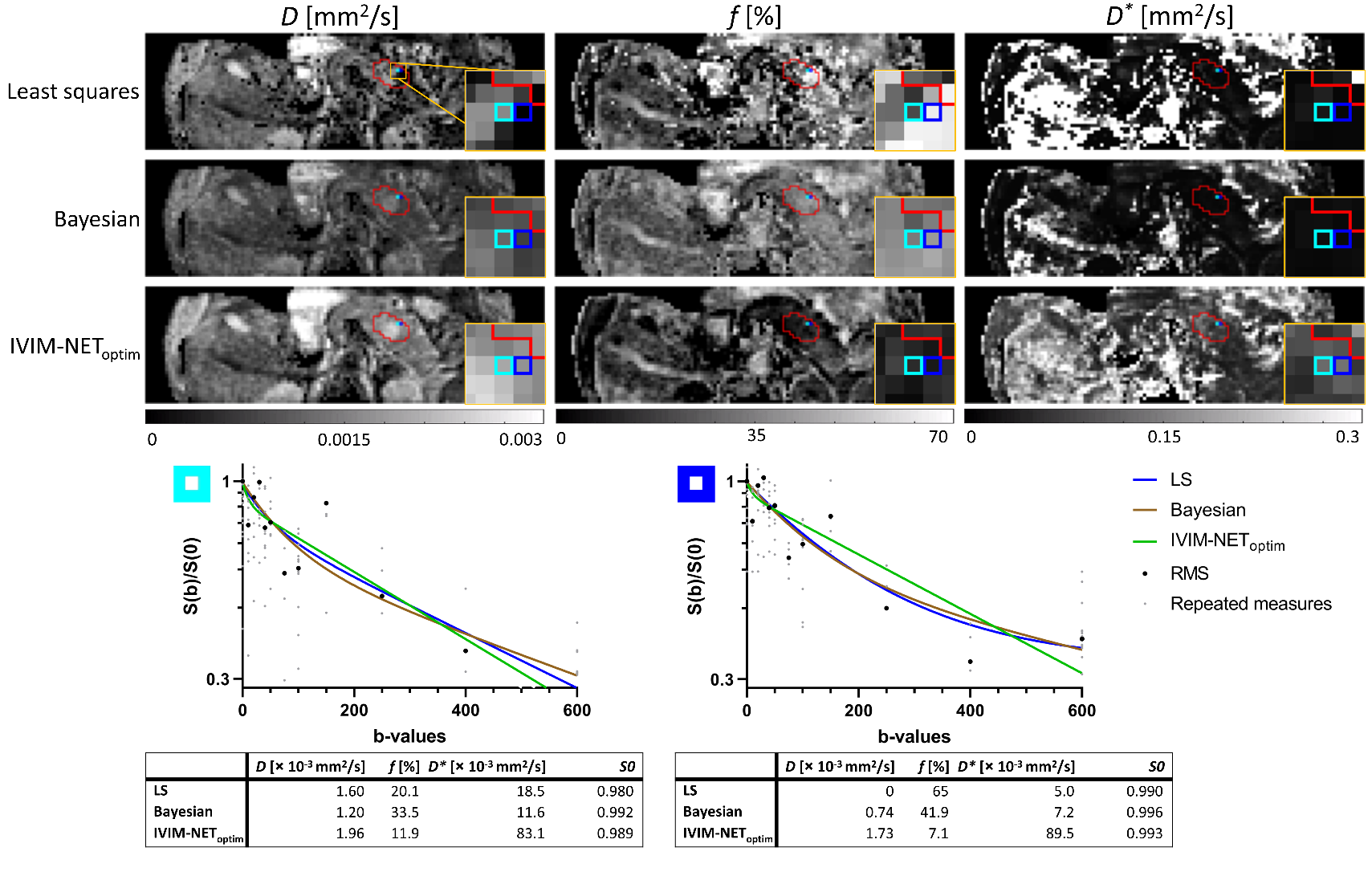


Figure S12: See Table S1.


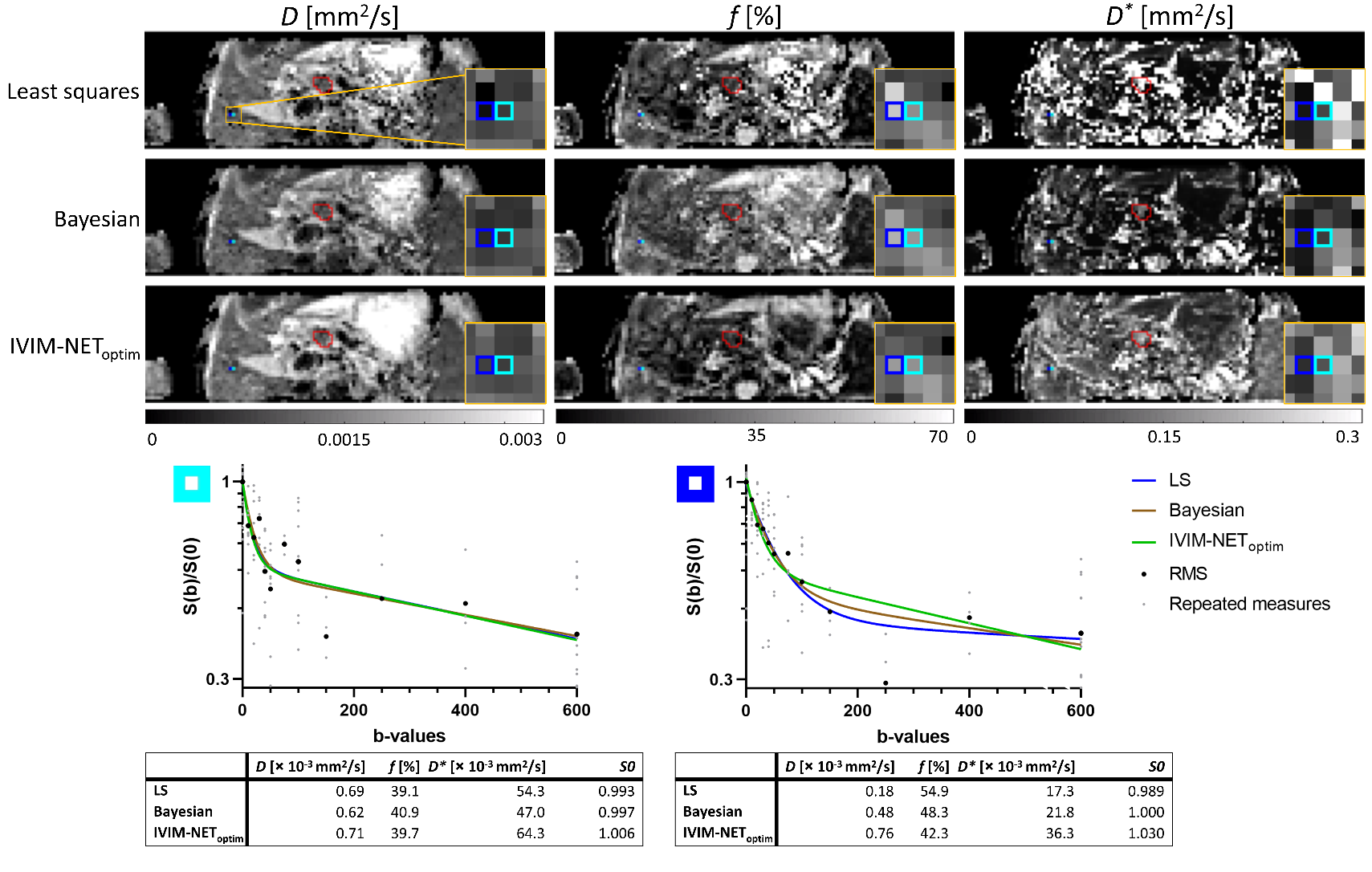


Figure S13: See Table S1.


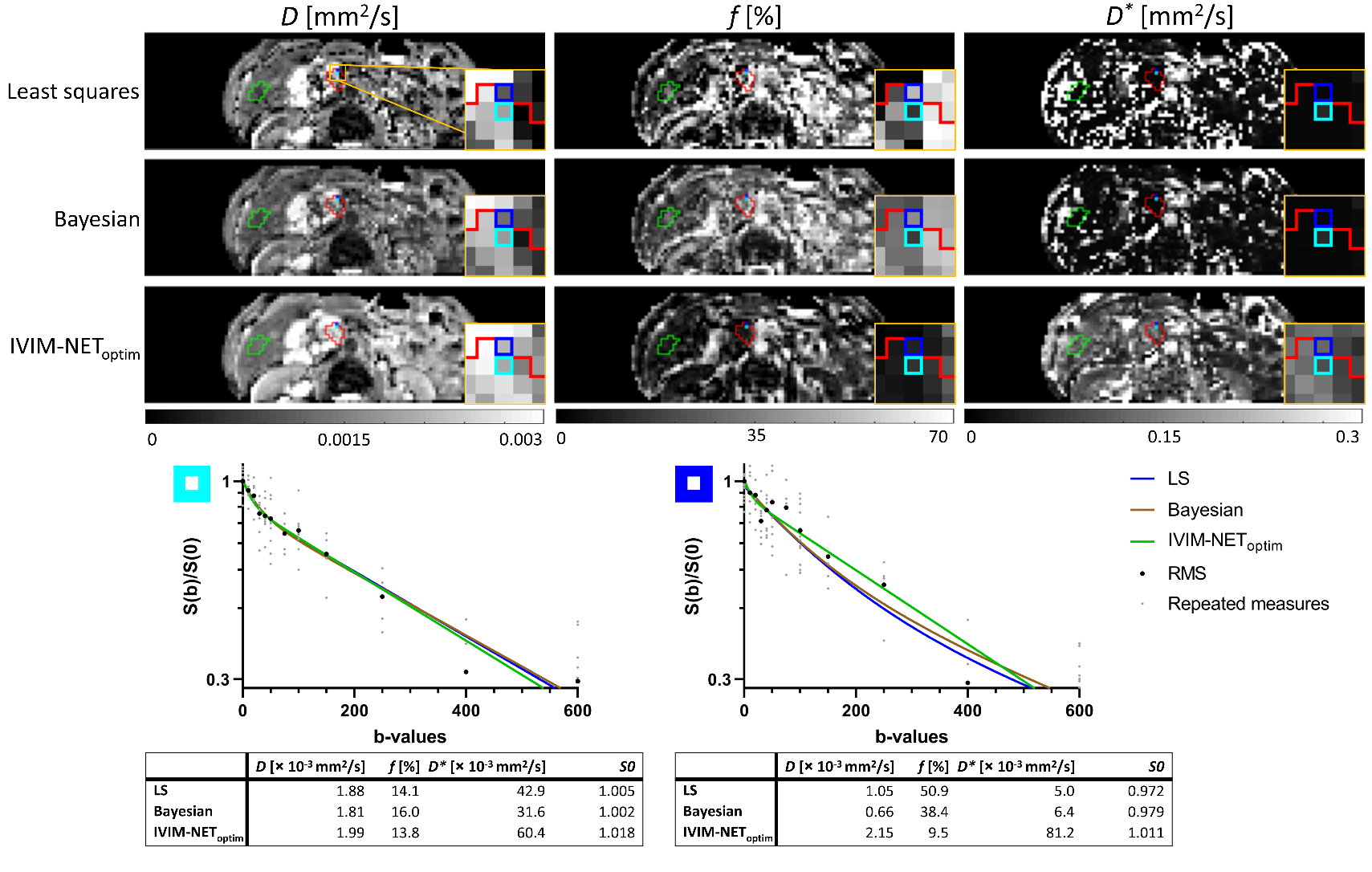


Figure S14: See Table S1.


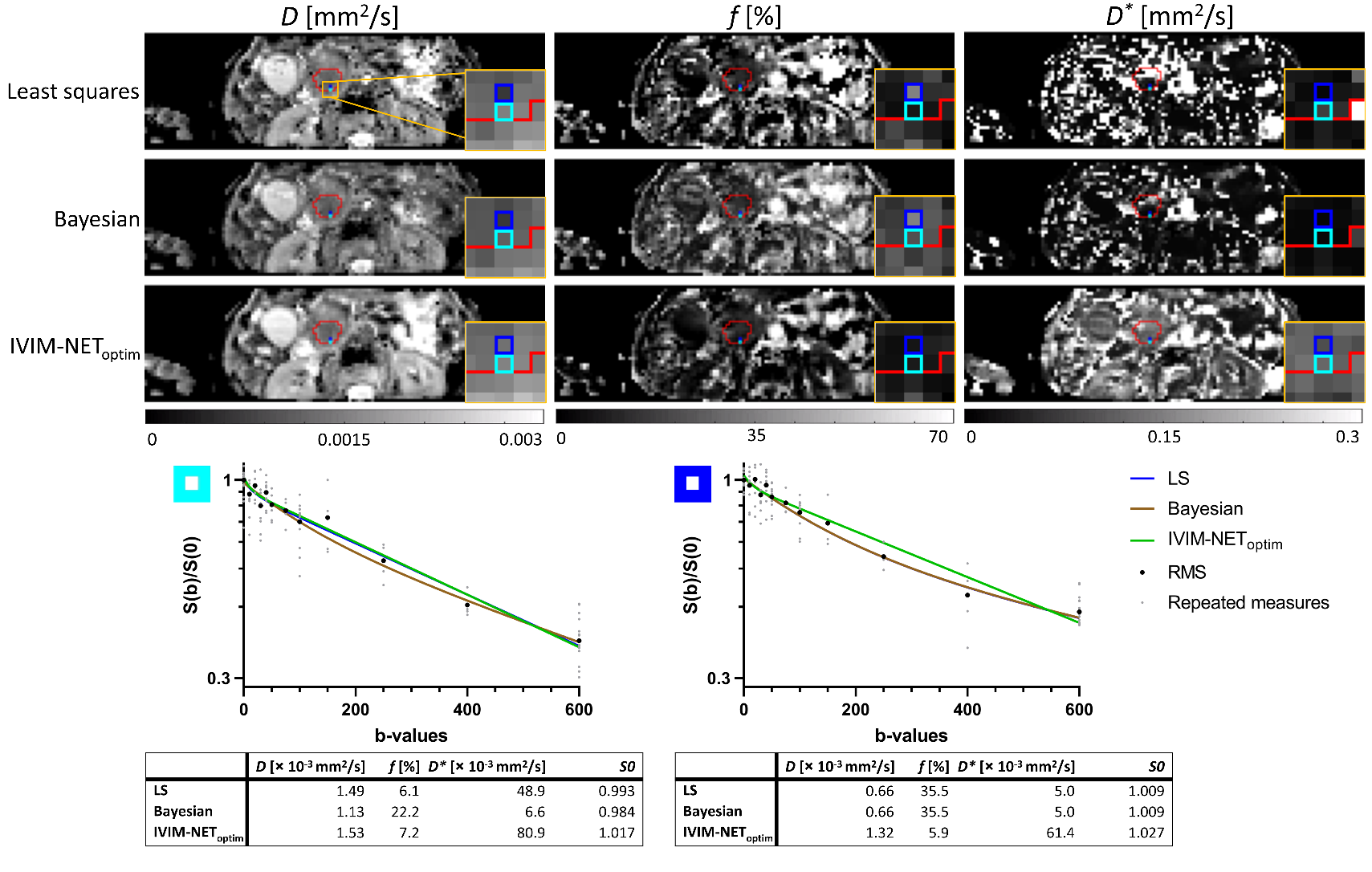


Figure S15: See Table S1.


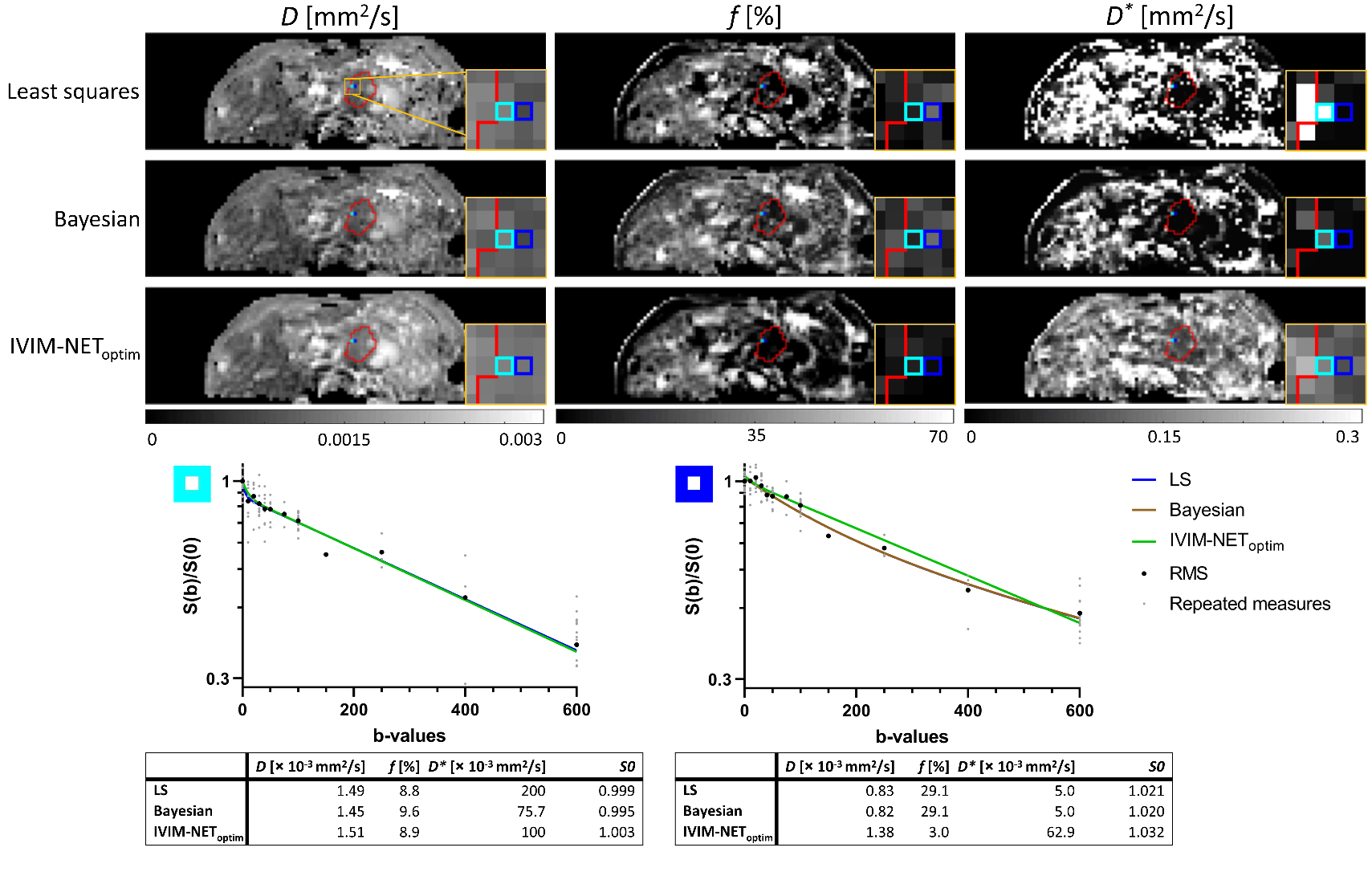


Figure S16: See Table S1.


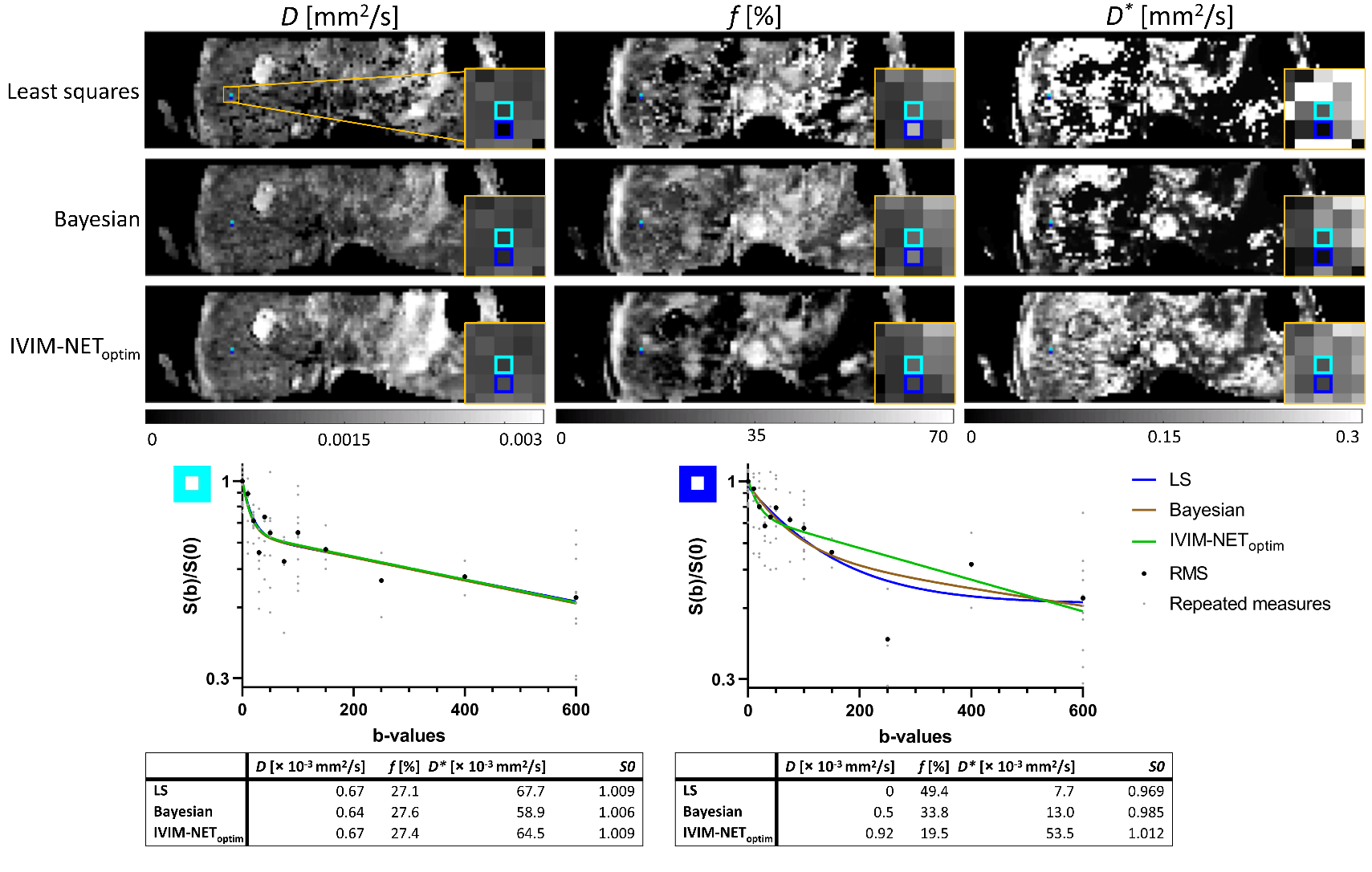


Figure S17: See table S1.


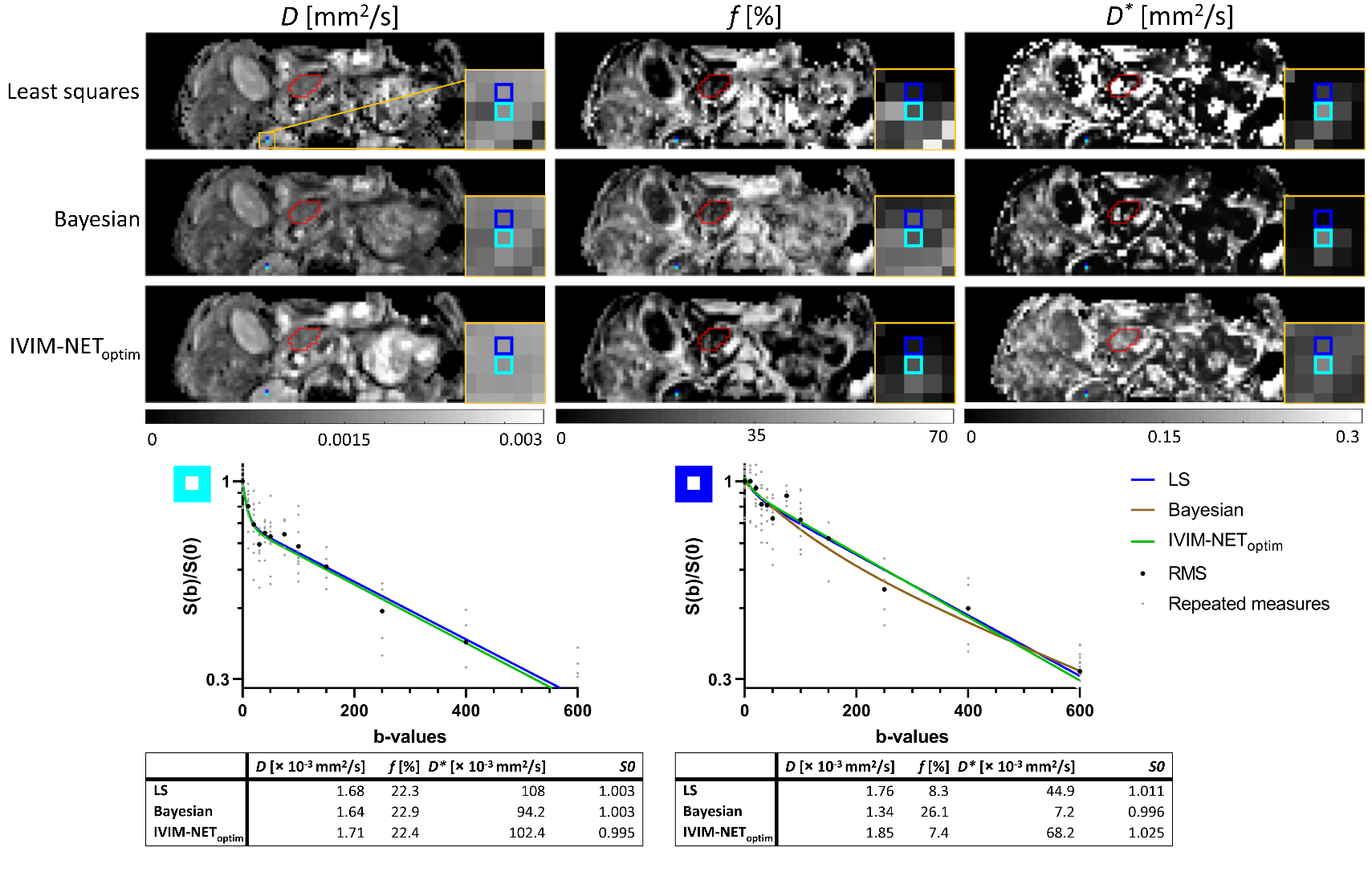


Figure S18: See Table S1.


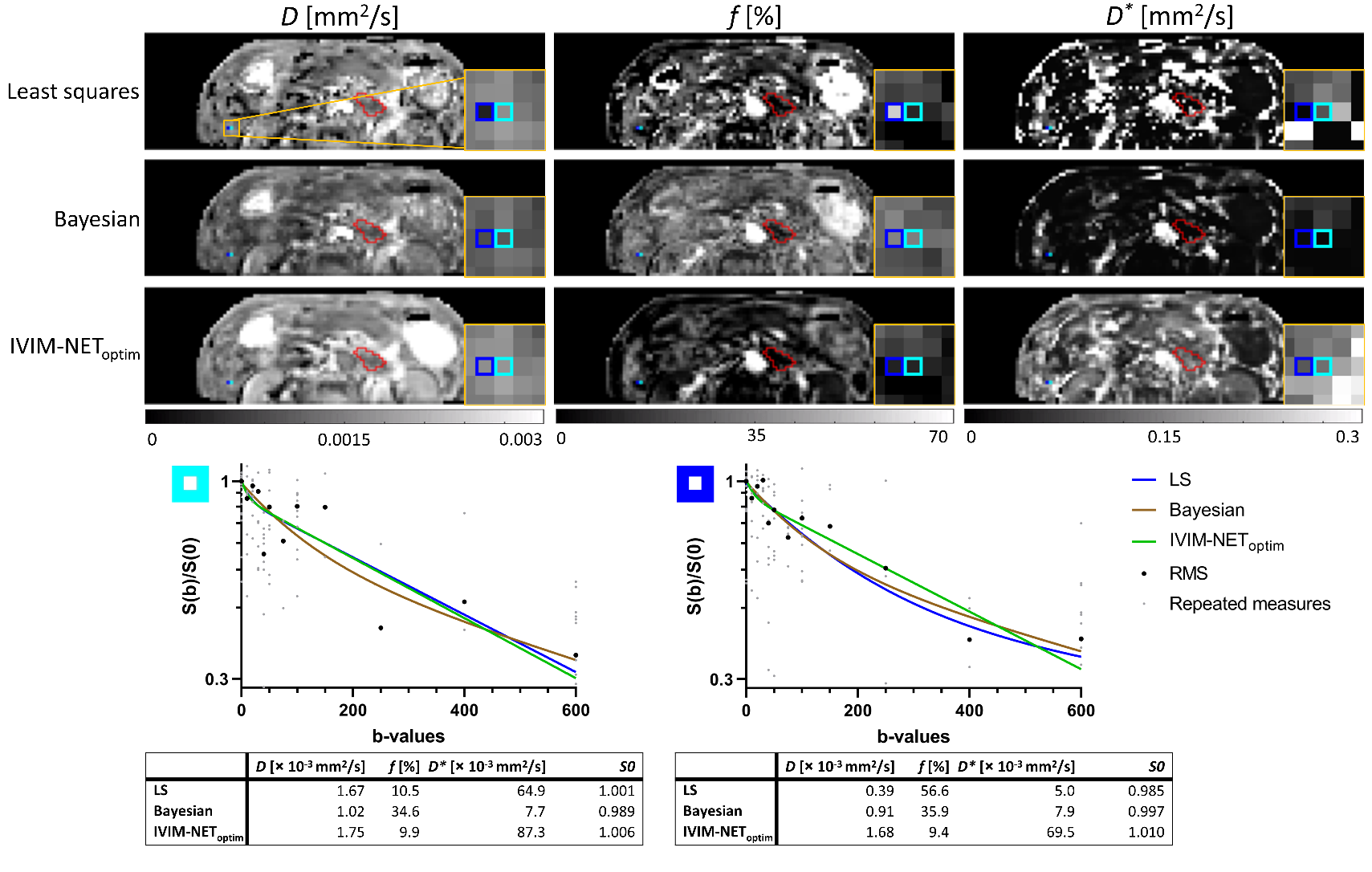


Figure S19: See Table S1.


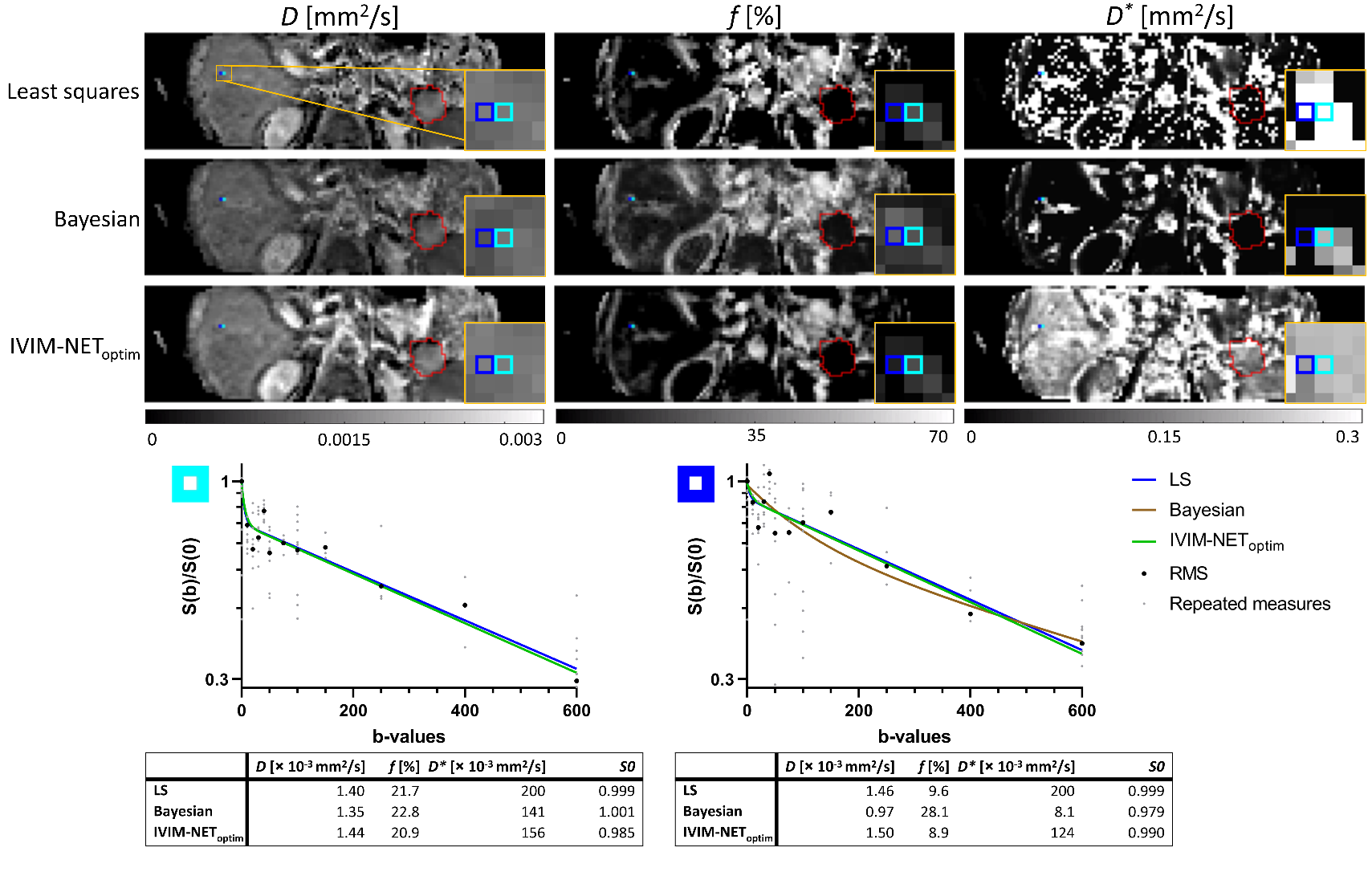


Figure S20: See Table S1.
